# An MRI-Compatible Foot-Sole Stimulation System Enabling Characterization of the Brain Response to Walking-Related Tactile Stimuli

**DOI:** 10.3389/fnins.2019.01075

**Published:** 2019-10-17

**Authors:** Tingwei Zhang, Kai Zhang, Junhong Zhou, Yufeng Chai, Yunfei Long, Xiaoying Wang, Brad Manor, Jue Zhang, Jing Fang

**Affiliations:** ^1^Academy for Advanced Interdisciplinary Studies, Peking University, Beijing, China; ^2^Hinda and Arthur Marcus Institute for Aging Research, Hebrew SeniorLife, Roslindale, MA, United States; ^3^Department of Medicine, Beth Israel Deaconess Medical Center, Boston, MA, United States; ^4^Harvard Medical School, Boston, MA, United States; ^5^College of Engineering, Peking University, Beijing, China; ^6^Department of Radiology, Peking University First Hospital, Beijing, China

**Keywords:** somatosensory, foot-sole stimulator, walking, fMRI, pneumatic, MRI-compatible

## Abstract

Foot-sole somatosensory impairment is a main contributor to balance decline and falls in aging and disease. The cortical networks involved in walking-related foot sole somatosensation, however, remain poorly understood. We thus created and tested a novel MRI-compatible device to enable study of the cortical response to pressure stimuli applied to the foot sole that mimic those stimuli experienced when walking. The device consists of a dual-drive stimulator equipped with two pneumatic cylinders, which are separately programed to apply pressure waveforms to the entire foot sole. In a sample of nine healthy younger adults, the pressure curve applied to the foot sole closely correlated with that experienced during over ground walking (*r* = 0.811 ± 0.043, *P* < 0.01). MRI compatibility testing indicated that the device has no or negligible impact on MR image quality. Gradient-recalled echo-planar images of nine healthy young adults using a block-designed 3.5-min walking-related stimulation revealed significant activation within the supplementary motor area, supramarginal gyrus, paracingulate gyri, insula, precentral gyrus, middle temporal gyrus, and hippocampus (uncorrected *P* < 0.001, *k* ≥ 10). Together, these results indicate that this stimulation system is MRI-compatible and capable of mimicking walking-related pressure waveforms on foot sole. It may thus be used as a research tool to identify cortical targets for interventions (e.g., non-invasive brain stimulation) aimed at enhancing this important source of input to the locomotor control system.

## Introduction

The decline in foot-sole somatosensation is highly prevalent in older adults and is a primary contributor to diminished gait and increased risk of falling (Richardson and Hurvitz, 1995; Roll et al., 2002; Hijmans et al., 2007; Kars et al., 2009; Manor et al., 2010). There are widely distributed skin receptors on the plantar surface of the foot sole, such as Merkel nerve endings, sensitive to the force, and distribution of contact pressures (Kennedy and Inglis, 2002; Halata et al., 2003). When walking, the foot soles are the only part of the body in direct contact with the ground, the skin receptors in the foot sole perceive the ground reaction forces during the stance phase of the gait cycle, and provide afferent input to the central nervous system in the regulation of walking. This input is not only involved in sub-cortical reflex loops, but is also delivered to cortical networks of the brain where it is integrated with other sensory inputs and used to form volitional movements during walking (Deliagina et al., 2008). However, the characteristics of the brain cortical networks pertaining to the regulation of this walking-related foot-sole somatosensation are not well understood.

Functional magnetic resonance imaging (fMRI) enables non-invasively measuring the neural activity in response to a given stimulus by quantifying the intensity of blood oxygenation level-dependent (BOLD) signal (Kwong et al., 1992; Glover, 2011). It requires people to remain motionless, however, and thus is not applicable to measure the characteristics of the brain during walking. Recently, several types of MRI-compatible mechatronic devices have been developed to stimulate foot during the fMRI scan. Gallasch et al. (2006) proposed a stationary moving magnet actuator to contact and vibrate the sole of foot with 20–100 Hz oscillations and 20 N maximum contact force to study cerebral responses evoked from mechanoreceptors. Hao et al. (2013) created and validated a pneumatic tactile stimulator applying programmable single-point pressure stimuli to a small area on the plantar surface of the foot. Such devices have proven to effectively activate several cortical regions, as measured by the change of intensity of BOLD signal. However, these previous studies focused on low-force and high-frequency stimuli, and stimulated only small surface of the foot sole, which therefore cannot enable the recreation of the pressure-waveform change on foot soles as experienced when walking over the ground.

In this study, we created a novel MRI-compatible foot-sole stimulation system, which is capable of applying controlled dynamic pressure waveform-type stimuli to the foot soles that mimic those experienced when walking, when the person lies motionless in the scanner. We completed two separate tests to (1) determine the similarity between real foot sole pressures experienced when walking and those simulated by the stimulation system, and (2) establish the effects of using this stimulation system on the quality of MR images. We then (3) completed a pilot study to explore the activation of the cortical networks in healthy younger adults, by assessing by the fMRI BOLD signal, when applying this stimulation system to the foot soles.

## Materials and Methods

### Foot-Sole Stimulation System Design

**FIGURE 1:**
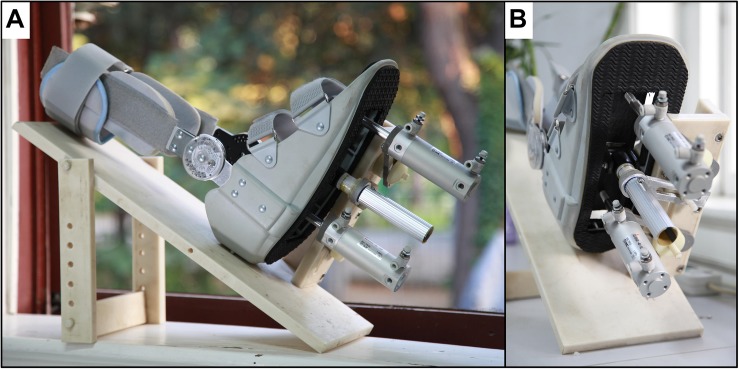
**(A)** The dual-drive foot-sole stimulator. **(B)** The executive unit of the stimulator.

We designed a foot-sole pressure stimulation system (Figure 1) to enable the simulation of the pressure waveforms as experienced by the foot sole during the stance phase of the gait cycle. The stance phase typically progresses from the heel (i.e., heel strike), to the ball of the foot (i.e., foot flat) and then the toe (i.e., toe-off) (Tiberio, 1987).

**FIGURE 2:**
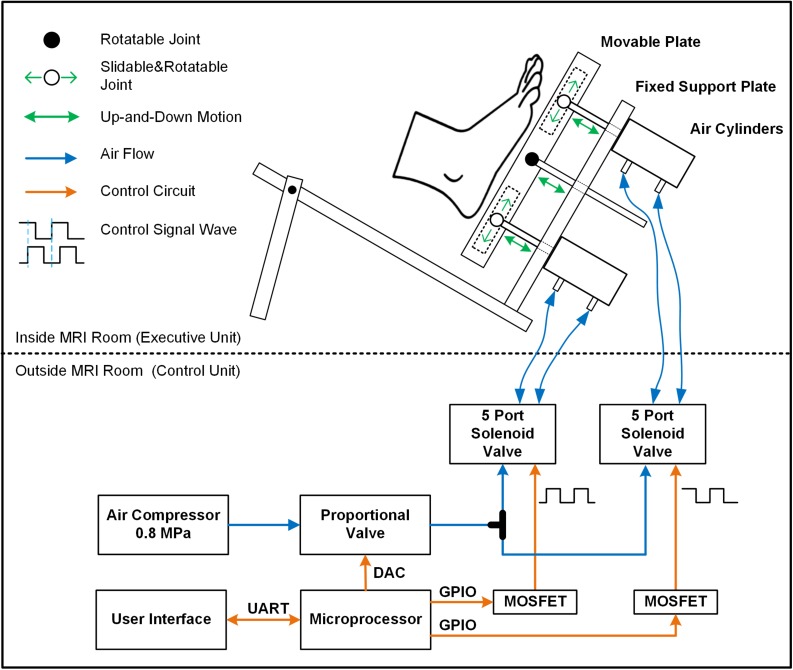
The diagram of the entire foot-sole pressure stimulation system. The control unit regulated the pressure by controlling the proportional valve and five-port solenoid valves following the pre-designed control signal waves (orange line). Airflow (blue line) generated from air compressor went through the designed routine to provide force to air cylinders. The pressure is applied to the foot sole by a rigid plastic movable plate board, which was actuated by two non-ferromagnetic aluminum air cylinders on the support platform. The middle item between two air cylinders was a support device to secure the stableness of the movable plate during the simulation.

This system included an air-compressor (GL0205, Greeloy, Shanghai, China) as the source of pressure, an execution unit, and a control unit (Figure 2). The execution unit (Figure 2, upper panel) consisted of two air cylinders (CG1BN32-40-XC6, SMC, Tokyo, Japan), a rigid plastic movable plate and a support platform. The two air cylinders were installed on the fixed support plate and respectively attached to a translatable and rotatable joint inside the movable plate so that they can actuate the plate asynchronously. The plastic movable plate pressed against the foot sole directly. To secure the plastic movable plate during the stimulating action, a rigid aluminum rod between the two cylinders was attached to a rotatable joint inside the movable plate through the fixed support plate. The entire execution unit was attached to a non-ferromagnetic (i.e., plastic and nylon materials) support platform, which is secured to the scanner table to limit the translation of applied pressures to the body and head. The control unit (Figure 2, lower panel) comprised two five-port solenoid valves (SY5120-5LZD-C6, SMC Corporation, Tokyo, Japan), a proportional valve (ITV2030-312L, SMC Corporation, Spain), a microcontroller (MSP430F168, Texas Instruments, Dallas, TX, United States), and a custom-developed user interface. A proportional valve was linked to the air compressor and the pressure of output airflow is controlled by the direct voltage (V_DC_) produced by digital to analog converter (DAC) of the microcontroller. The relationship between V_DC_ input to the proportional valve and the pressure of output airflow was linear, enabling precise control of the magnitude of applied pressure. The proportional valve transfers output airflow to the two five-port solenoid valves through a tee coupling, and these two valves control two air cylinders separately to produce one-degree-of-freedom (DOF) oscillations by shaping the airflow following the control signal wave sequences. The signal wave sequences were pre-programed and produced by the GPIOs (General-Purpose Input/Output) of the microcontroller. The custom-developed user interface is based on Matlab (The MathWorks, Inc., Natick, MA, United States) and able to communicate with the microcontroller through UART (i.e., universal asynchronous receiver-transmitter). The V_DC_ and frequency of control signal wave sequences produced by microcontroller can be configured in real-time easily by user. By doing so, the microcontroller can regulate the magnitude, frequency, and sequence of pressure applied to the foot sole following the configuration.

According to the configuration, the cylinder can then reverse its movement direction within 100 ms, enabling a maximal oscillatory frequency of 10 Hz. The output force of this stimulator ranged from 5 to 500 N approximately, and the speed of the movable plate’s movement is between 40 and 1000 mm/s. When using this stimulator within the MRI setting, the air compressor and control unit were located outside the scanner room and concatenated with the two air cylinders inside the scanner room via four 5 m-long and 6 mm-diameter high-pressure polyurethane tubes.

As shown in Figure 1, we also modified and installed a medical ankle joint support brace on the support platform, which is used to secure the shin and make the foot sole face the movable plate passively. The angle of knee and hip joint is adjustable based upon the comfort of each participant. We chose to fix the ankle joint at 90° of dorsiflexion to minimize head movements during stimulation following the previous study (Hao et al., 2013).

### Study Protocol

#### Experiment 1: Simulation of Walking-Related Foot-Sole Stimuli Using the Foot-Sole Stimulator

We first examined the capacity of our stimulation system to apply pressures to the foot sole that mimic those experienced when walking, by comparing the pressures generated by the stimulation system to actual pressures experienced during walking.

##### Participants

Nine healthy young participants aged 20–29 years with right-foot dominance were recruited and provided written informed consent as approved by the local ethical committee. Exclusion criteria included any acute illness, self-reported history of cardiovascular, metabolic, or neurological disease, musculoskeletal disorders, major foot deformity or history of surgery or major injury to the lower extremities.

##### Test procedure

Each participant completed four trials within each of the following tests: walking test and stimulating test. We used an instrumented foot pressure insole (F-scan 3000E, Tekscan, United States) to measure the pressure on the foot soles in the two tests.

In the walking test, the insole was inserted into the participant shoes to record pressure waveforms during four trials of straight walking at preferred speed. Participants performed at least four complete gait cycles within each trial.

**FIGURE 3:**
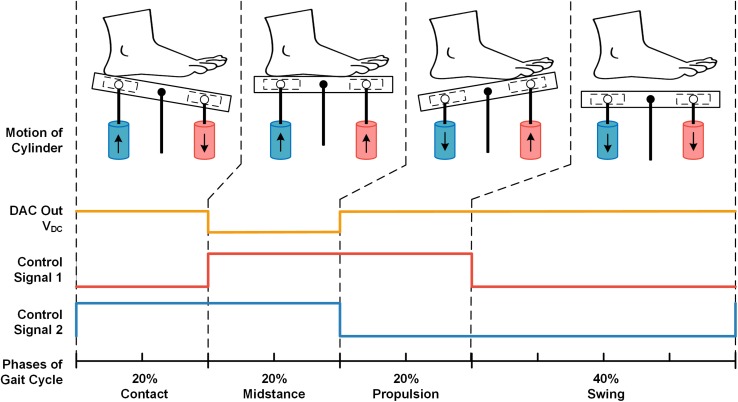
The example of pre-designed signals from two cylinders to mimic pressures in one complete gait cycle. The V_DC_ signal produced by DAC (orange line) regulates the output pressure force of the cylinder. The control signal 1 (red line) controls the up-and-down motion of the red cylinder, and the control signal two (blue line) controls the blue cylinder’s motion (i.e., Logical high corresponds to up-motion, logical low corresponds to down-motion). There were four states in one period of the control signal waves (i.e., LH, HH, HL, and LL), corresponding to the four phases of one stimulating gait cycle: contact, midstance, propulsion, and swing.

In stimulating test, we programed the force of pressure in midstance phase to equal the bodyweight of each participant. The frequency of applied pressure stimuli was configured following the frequency of the walking pattern. The force control signal V_DC_ and two motion control signal waves were shown in Figure 3. Lower V_DC_ signal was programed in the midstance phase compared to other gait phases, since the force in the contact and propulsion phases of gait is oftentimes greater than the force in stance phase (i.e., body weight) during walking. To match the gait phases of general walking, the time ratios of the four stimulating phases over the entire gait cycle were set as: contact-20%, midstance-20%, propulsion-20%, and swing-40% (Perry et al., 1992). We used dual adhesive tape to fix the pressure sensor of insoles on the movable plate surface to record the pressure applied on the right foot sole by the stimulator. When participants were lying supine (similar to their posture in MRI scanner), the foot sole was stimulated at least four times in each trial. All participants were told to relax their lower limbs during the test.

#### Experiment 2: MRI Compatibility Test

To verify the influence of the stimulator to the MRI scanner stability, following the fMRI Quality Assurance protocol (Friedman and Glover, 2006) and the previous study (Hao et al., 2013), we then measured the signal-to-noise ratio (SNR), signal-to-fluctuation noise ratio (SFNR) and magnetic field map in MRI scan by imaging a water phantom in each of the following conditions. (1) Power-on: foot-sole stimulator was working in only 100 cm (closer than the distance when the participants in scanning) away from the phantom center in MR scanner room. (2) Power-off: the stimulator was 100 cm away from the phantom center in the MR scanner room but not working. (3) Absent from MRI: foot-sole stimulator was out of the MR scanner room.

##### MRI scan

The MR imaging was performed on a 3T scanner (Discovery MR750, GE Healthcare, Milwaukee, WI, United States) using an eight-channel head coil. The Acquisition parameters were shown in Table 1.

**TABLE 1:** List of acquisition parameters in MRI compatibility test.

**Parameter**	**Functional**	**Field map**
Sequence type	Gradient-recalled echo-planar imaging	2D fast spoiled gradient echo
Scan plane	Axial	Axial
Repetition time (TR)	2000 ms	488 ms
Echo time (TE)	30 ms	2.5 ms/5.8 ms
Field of view	23 cm × 23 cm	23 cm × 23 cm
Flip angle	90°	60°
Matrix size	64 × 64	256 × 256
Number of slices	28 interleaved axial slices	15
Slice thickness	4 mm with 1 mm spacing	3 mm with 1 mm spacing
Number of volumes	200	1

##### Data analysis

All images in each condition were processed using SPM8 (the Wellcome Trust Centre for Neuroimaging, London, United Kingdom) (Tzourio-Mazoyer et al., 2002) and custom programing with MATLAB. In addition to a visual inspection of artifacts, we quantified the image quality using three types of parameters: SNR, SFNR, and magnetic field map. A 21 × 21 voxel region-of-interest (ROI) placed in the center of the image was created. The SNR parameters were measured following the methods delivered by the National Electrical Manufacturers Association (Friedman and Glover, 2006; National Electrical Manufacturers Association [NEMA], 2008); The SFNR of functional images was calculated following the fMRI Quality Assurance protocol (Friedman and Glover, 2006). The magnetic field maps were estimated based on EPI, and the local resonance frequency shift of each voxel was calculated to check the field non-uniformities and the subtle magnetic field perturbations potentially arising from the presence of the stimulator (Reber et al., 1998; Windischberger et al., 2004; Hao et al., 2013).

#### Experiment 3: Brain Activation Test

##### Participants

Nine healthy young participants (6 males, 3 females, 23 ± 3 years, 61 ± 10 kg, and 171 ± 10 cm) were included in this test. All of them signed written informed consent approved by the local ethical committee. Inclusion criteria were right-foot dominance; the ability to perceive 10 g of pressure at five weight-bearing sites on the right foot sole as determined with a 5.07-gauge Semmes–Weinstein monofilament; and the preferred walking speed faster than 1.0 m/s. Exclusion criteria included any known neurological or musculoskeletal disorders, previous surgery on the back or lower extremities and contraindication for MRI.

##### fMRI scan

**FIGURE 4:**
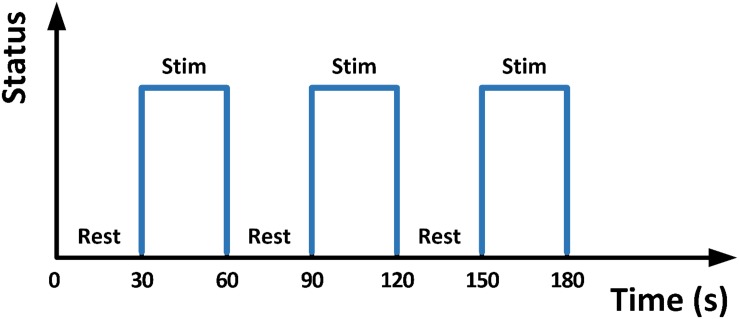
The 30 s Stim-Rest block designed stimulation.

A gradient-recalled echo-planar imaging (GRE-EPI) sequence was utilized in the same 3T MRI machine. Acquisition parameters were: TR = 2000 ms, TE = 30 ms, flip angle = 90°, matrix = 64 × 64, thickness/spacing = 4 mm/1 mm, field of view (FOV) = 24 × 24 cm, and 33 interleaved axial slices. The maximum stimulation pressure was set equal to 10% of the body mass of each participant, as determined by a dynamometer (NK-500, AIPU, Anhui, China). All participants were barefooted and instructed to relax their lower limbs. A block-designed 3.5-min stimulation protocol consisting of alternating blocks of 30 s-Rest (i.e., no stimulation) and 30 s-Stim (Figure 4) was applied to the right foot sole during fMRI scan. During 30 s-Stim, the stimulator applied 1 Hz square wave with 50-percent duty cycle and 90° phase difference to the two actuators, thus could alternately press the participants’ front sole and heel, mimicking the typical ground reaction force experienced when walking.

##### Data analysis

SPM8 was applied to calculate functional activation maps. To correct for potential head movement between scans, images were realigned with the first scan image and compensated delays associated with acquisition time differences via time correction. Six-parameter head motion curves were obtained. A 2 × 2 × 2 mm^3^ Montreal Neurological Institute template was applied to normalize all images. Activation patterns for each individual were detected by general linear modeling. With the full width/half maximum parameter set to 8 mm and temporally filtered using a cutoff of 128 s, functional images were spatially smoothed using a Gaussian filter. Head motion was monitored by head motion parameters in SPM8. The head motion was significantly higher in the *Z*-axis (i.e., the direction of applied pressure) compared to the other directions, but was still less than 1 mm.

### Statistical Analysis

All the statistics were performed by using IBM SPSS Statistics (IBM, Inc., United States) and custom MATLAB programing (The MathWorks, Inc., Natick, MA, United States).

#### Experiment 1

We obtained four walking pressure curves and four stimulating pressure curves from each of the participants. Focusing on the similarities in one gait cycle, we split a one-gait-cycle pressure curve from each of the eight pressure curves by using the Footscan Insole Software (Version2.39, Tekscan, United States) for the following analysis. To determine the correlation (i.e., similarity) between the pressure of walking and stimulating, we calculated 16 Spearman’s correlation coefficients between the four one-gait-cycle walking pressure curves and four one-gait-cycle stimulating pressure curves. Significant level was set at *P* < 0.01. To assess the difference between the magnitude of pressure in walking and stimulating, we calculated the percent difference (PD) of pressures in three exertion phases: contact, midstance, and propulsion. The PD was defined as (MSP–MWP)/MWP, in which the MSP was mean of stimulating pressure and the MWP was mean of walking pressure. For comparison of pressure maps, we obtained five pressure maps (i.e., in contact phase, midstance phase, propulsion phase, swing phase, and complete gait cycle) from pressure records of walking and stimulating, respectively. The trajectory points of gravity center during each phase were also calculated.

#### Experiment 2

To test the effect of the device on the image quality, we performed a one-way analysis of variance (ANOVA). Model effects were the testing conditions (i.e., device power-on, power-off, and absent from MRI, and the dependent variables were the image quality parameters (i.e., SNR, SFNR, and magnetic field map).

#### Experiment 3

We applied one-sample *t*-tests to generate a group result on the t-map of each individual (uncorrected *P* < 0.05, at least 10 contiguous voxels).

## Results

### The Pressure Applied by Stimulator Was Correlated to That Experienced During Walking

**FIGURE 5:**
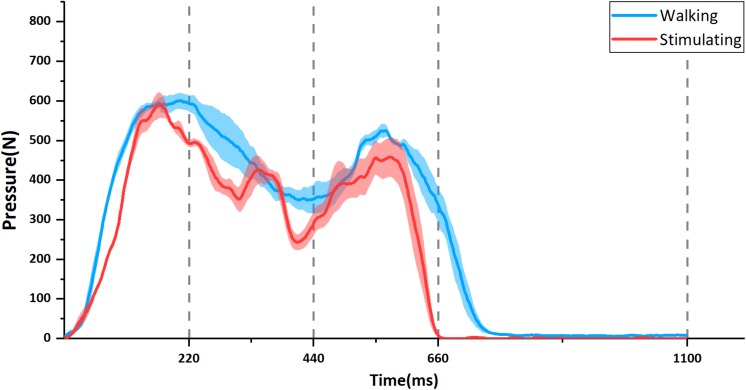
The example of temporal change in foot-sole pressure in one participant. The blue line is the change during walking and the red is when stimulated by the stimulator. Each curve is the mean value with standard errors (shadow) from four separate pressure curves. Corresponding to the four states of control signal waves shown in Figure 3, the time axis is divided into four phases: Contact (0–220 ms), Midstance (220–440 ms), Propulsion (440–660 ms), and Swing (660–1100 ms).

Figure 5 showed one example of participant’s gait-cycle pressure curve. The two curves are the mean value with standard error bars of four pieces of real walking pressure curve (blue line) and stimulating pressure curve (red line), respectively. Both of them had similar trend and fluctuation. Particularly, the pressure peaks in the heel contact phase and propulsion phase occurred in both two curves. All other participants’ pressure curves are available in the Supplementary Materials (Supplementary Figure S1).

**FIGURE 6:**
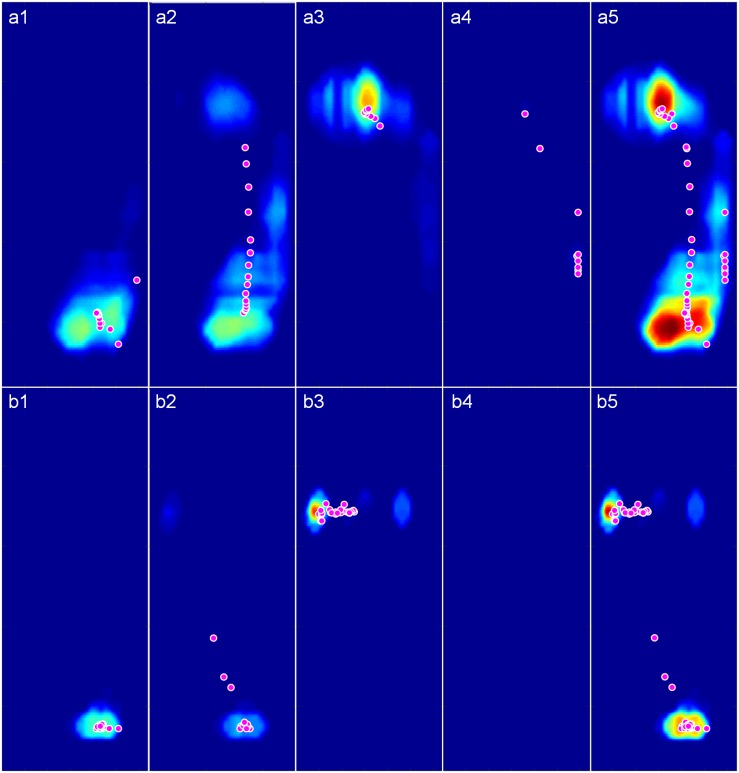
The pressure maps with trajectory points of gravity center (Magenta circles with white edge) during walking and stimulating. **(a1–a4)** Pressure maps in contact, midstance, propulsion, and swing phase of a complete walking gait cycle. **(b1–b4)** Pressure maps in the four phases of a complete stimulating gait cycle. **(a5)** Pressure map of the whole walking gait cycle. **(b5)** Pressure map of the whole stimulating gait cycle.

The participant’s pressure maps with trajectory points of gravity center (red circles) during walking and stimulating were shown in Figure 6. In contact phase, the plantar pressure distribution in stimulating first appeared on the heel (Figure 6b1) and spread to the anterior foot sole during mid-stance phase (Figure 6b2), then focused on the ball of foot in propulsion phase (Figure 6b3) and disappeared in the swing phase (Figure 6b4), similar to the pattern of pressure distribution changes during walking (Figures 6a1–a4). The higher-pressure values in both contact and propulsion phases were consistent with the two pressure peaks shown in the pressure curves (Figure 5). During the whole gait cycle, the gravity center transferred from heel to ball of foot both in walking and stimulating, the difference is that the trajectory points tended to a straight line in stimulating (Figure 6b5, red circles), while in walking, the trajectory points protruded to the right lateral (Figure 6a5, red circles). Other eight participants’ pressure maps were shown in the Supplementary Materials (Supplementary Figures S2–S9).

The results of Spearman’s correlation coefficient analyses showed the programed pressure was significantly correlated with the actual pressure as experienced during walking (*r* = 0.811 ± 0.043, *P* < 0.01, Table 2). The results of mean pressure difference ratio of all nine participants showed that the stimulating pressures were lower than the walking pressures (−16.76 ± 6.33% in contact phase, −16.64 ± 8.00% in midstance phase, −17.80 ± 5.57% in propulsion phase, Table 3).

**TABLE 2:** Mean and standard deviation of 16 (4 walking trials with 4 stimulating trials) Spearman’s correlation coefficients of each participant.

**Participant**	**1**	**2**	**3**	**4**	**5**	**6**	**7**	**8**	**9**	**Average**
Mean	0.800	0.746	0.829	0.711	0.899	0.725	0.899	0.805	0.887	0.811
SD	0.084	0.067	0.049	0.041	0.018	0.029	0.023	0.034	0.046	0.043

*SD, standard deviation. The detailed results were shown in Supplementary Table S1.*

**TABLE 3:** Percent difference (PD) of mean stimulating pressure compared to mean walking pressure in contact, midstance and propulsion phase of each participant.

**Participants**	**PD in contact/%**	**PD in midstance/%**	**PD in propulsion/%**
1	–22.87	22.14	–36.24
2	–22.56	–12.17	2.51
3	–38.95	11.07	–25.87
4	–10.37	–27.88	–12.14
5	1.01	–56.17	–9.88
6	–45.83	–38.57	8.13
7	3.26	–7.72	–23.78
8	–14.49	–15.83	–20.60
9	–20.80	–24.67	–42.36
Mean	–16.76	–16.64	–17.80
Standard error	6.33	8.00	5.57

*PD, percent difference = (mean stimulating pressure – mean walking pressure)/mean walking pressure.*

### Compatibility of the Foot-Sole Pressure Simulator With 3T MRI

**FIGURE 7:**
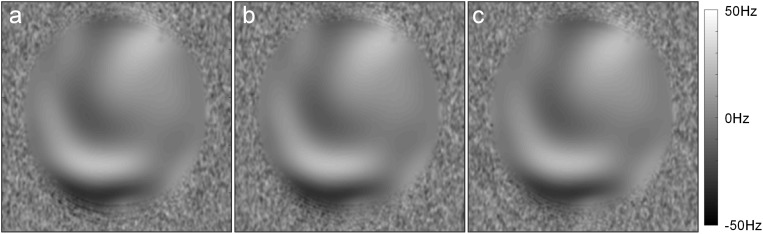
The field maps tested in the three conditions: **(a)** Power-on, **(b)** Power-off, **(c)** absent from MRI.

Images of the phantom demonstrated that the foot sole stimulator had no impact on the quality of MR images. Specifically, visual inspection revealed no observable differences between conditions (device power-on, power-off, and absent from MRI). Within the tested ROI of images, no significant differences were observed among the SNR parameters (*P* = 0.82, *F* = 0.198, *F*crit = 3.03) and the SFNR parameters (*P* = 0.43, *F* = 0.845, *F*crit = 3.00) of functional images (Table 4). The results of field mapping test were shown in Figure 7, the field non-uniformities under the three testing conditions were all less than ±50 Hz, and there was no observable differences in visual inspection among the conditions.

**TABLE 4:** MRI compatibility test on phantom center ROI.

**Stimulator**	**SNR**	**SFNR**
**condition**	**(Mean ± SD)**	**(Mean ± SD)**
Power-on	29.43 ± 3.25	631 ± 51
Power-off	29.69 ± 3.63	627 ± 52
Absent from scanner	29.71 ± 3.52	630 ± 45

*SNR, signal-to-noise ratio; SD, standard deviation; SFNR, signal-to-fluctuation noise ratio.*

### Cortical Response to Foot-Sole Pressure Simulator

**FIGURE 8:**
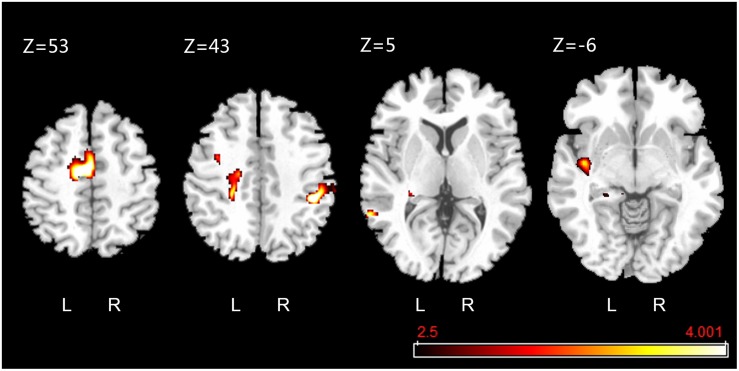
Active clusters overlaid on a standard T1 template obtained during foot-sole pressure simulation compared to rest. Walking-related pressure stimuli applied to the right foot sole simulation was associated with increased BOLD signal intensity within the pre-motor and supplementary motor cortex (SMA.L, *Z* = 53), precentral gyrus (PreCG.L, *Z* = 43), median cingulate and paracingulate gyri (DCG.L, *Z* = 43), supramarginal gyrus (SMG.R, *Z* = 43), middle temporal gyrus (MTG.L, *Z* = 5), hippocampus (HIP.L, *Z* = 5), and insula (INS.L, *Z* = –6) (uncorrected *P* < 0.001, *k* ≥ 10).

The fMRI results showed that, compared to the rest condition, the intensity of BOLD signal (i.e., excitability) of supplementary motor area in left medial frontal gyrus, supramarginal gyrus of right inferior parietal lobule, median cingulate and paracingulate gyri, left insula, precentral gyrus, middle temporal gyrus, and hippocampus of left parahippocampal gyrus were significantly increased (uncorrected *P* < 0.001, *k* ≥ 10) (Figure 8). Besides, we did not visually detect arresting motion artifacts or deviant statistical parameters during data processing.

## Discussion

We designed a novel foot sole stimulation system to enable the study of the cortical response to walking-related foot sole pressure stimuli. In this study, we demonstrated that: (1) the pressure waveforms applied to the foot soles provided by this dual-drive stimulation system closely mimicked those experienced during over-ground walking; (2) the use of this stimulation system has no interference with the quality of MR image; and (3) the walking-related foot sole stimuli activates a distributed functional network that includes multiple motor and somatosensory cortical regions.

The foot-sole pressure stimulation system we developed mimics the temporal change of pressure as experienced during over-ground walking. The map of pressure applied by the stimulator matched with that of actual walking, demonstrating that the stimulator is able to spatially simulate the pressure distribution on plantar to a certain extent. Compared to those previous MRI-compatible stimulators (Gallasch et al., 2006; Hollnagel et al., 2011; Hao et al., 2013; Hartwig et al., 2017) applying solely vibrotactile stimuli, here this stimulator delivers the pressures on the whole foot sole surface by using two separately controlled pneumatic cylinders to drive a large plate. Moreover, the pressure magnitude and stimulation frequency can be controlled separately to match stance-phase pressures of healthy participants with very small motion artifact to the MRI scan. Future studies are worthwhile to explore if this stimulator can simulate the walking pressure patterns in those with impaired mobility, such as those suffering from Parkinson’s disease.

Activation of multiple brain cortical regions, including the pre-motor and supplementary motor cortex (SMA.L) of the left medial frontal gyrus and precentral gyrus [i.e., primary motor cortex (MI)], left insula (INS.L), median cingulate and paracingulate gyri (DCG.L), supramarginal gyrus, middle temporal gyrus, and hippocampus, was observed in response to the walking-related pressure stimuli applied by this stimulator. Several studies (Seitz and Roland, 1992; Gallasch et al., 2006) observed the activation in SMA.L and MI in response to high-frequency vibratory stimuli and low-frequency, large-force pressure stimulation (Hao et al., 2013). The results of our study confirmed those results and provided evidence that these regions are pertaining to the regulation of pressures on the foot soles as experienced during walking over the ground. The activation within INS.L and DCG.L is consistent with previous studies applying the vibratory stimulation to foot soles (Golaszewski et al., 2002; Gallasch et al., 2006), revealing the insula and cingulate cortex play important role in somatosensory processing (Vogt et al., 1992); (Schneider et al., 1993). Future studies are needed to explore the underlying neurophysiology inside the activation of supramarginal gyrus, middle temporal gyrus, and hippocampus in response to the walking-related foot sole stimulation, which can be taken into account when designing the strategies to target the cortical regions for the restoration/improvement in foot-sole somatosensation.

It should be noted that the characteristics of each stride (e.g., length or time) are different between each other within one walking trial and between each walking trial. In this study, the pressure waveforms applied by the stimulator simulates the averaged tempo-spatial characteristics of strides across trials. Future studies are thus needed to introduce the cycle-to-cycle variation of pressures, enabling a more appropriate replication of walking-related pressure stimuli. The observed lower magnitude of stimulating pressure, compared to real ground-pressure during walking, maybe because the maximum output force of the air cylinder is limited and the fixation to foot was not stable enough to resist the exerted pressure. The center of gravity track tended to a straight-line during stimulation. The potential reason is that the pressure was limited in one degree of freedom, but during real walking, the ankle can also be flipped inward or outward to adjust the bearing area of foot sole. We investigated the brain’s response in a cohort of relatively small sample size by applying pressures of 10% of body mass. Future studies of larger sample size are thus needed to confirm the results of this pilot study and explore the effects of pressures with different intensity on excitability of brain regions. This study explored the brain’s response to the entire gait cycle. It will thus be worthwhile to explore such response to different gait phase separately in future studies, enabling a sophisticated understanding of cortical regulation in the walking. Meanwhile, regarding to the optimization of the stimulator, future studies can: (1) add a DOF perpendicular to the existing DOF to mimic the twist of ankle; (2) equip each air cylinder with a separate proportional valve to enable more smoother control for pressure output; and (3) mount multi-point pressure sensor on the movable plate and fed back the real-time pressure distribution to the controller, forming closed control loop for more accurate stimuli.

## Conclusion

This novel foot-sole stimulation system is feasible to mimic the pressure on foot soles as those experienced during walking on the ground and is compatible to be used during the MRI scan. The walking-related foot sole pressure stimuli applied by this system activated a distributed cortical network within the brain. Therefore, it can serve as a valuable tool stimulating personalized pressures to explore the characteristics of functional brain networks relating to the perception and modulation of foot-sole somatosensation during walking.

## Data Availability Statement

The datasets generated for this study are available on request to the corresponding author.

## Ethics Statement

This study was carried out in accordance with the recommendations of the Institutional Review Board of Academy for Advanced Interdisciplinary Studies at Peking University and the Institutional Review Board of Department of Radiology, Peking University First Hospital with written informed consent from all subjects. All subjects gave written informed consent in accordance with the Declaration of Helsinki. The protocol was approved by the Academy for Advanced Interdisciplinary Studies at Peking University and Department of Radiology at Peking University First Hospital.

## Author Contributions

TZ performed the pressure compare test, MRI compatibility test, and data analysis. KZ designed the stimulator and made the prototype. JnZ helped with experiment design, statistical analysis, and manuscript revising. YC provided indispensable guidance on the mechanical design and electrical control of the stimulation system. YL conducted the brain activation test and assisted fMRI data processing. XW provided the MRI scanner and gave valuable instruction for MRI data collection. BM, JeZ, and JF conceptualized the study and supervised the research. All authors contributed to the writing of the manuscript and have approved the final version of the manuscript.

## Conflict of Interest

The authors declare that the research was conducted in the absence of any commercial or financial relationships that could be construed as a potential conflict of interest.
