# Supplementary material for: An MRI-Compatible Foot-Sole Stimulation System Enabling Characterization of the Brain Response to Walking-Related Tactile Stimuli
Source: Front Neurosci. 2019 Oct 17;13:1075. doi: 10.3389/fnins.2019.01075 (PMC6811610; doi:10.3389/fnins.2019.01075)
Supplement: Supplementary file 1 [file Data_Sheet_1.docx]

Supplementary Material

1. Supplementary Figures and Tables


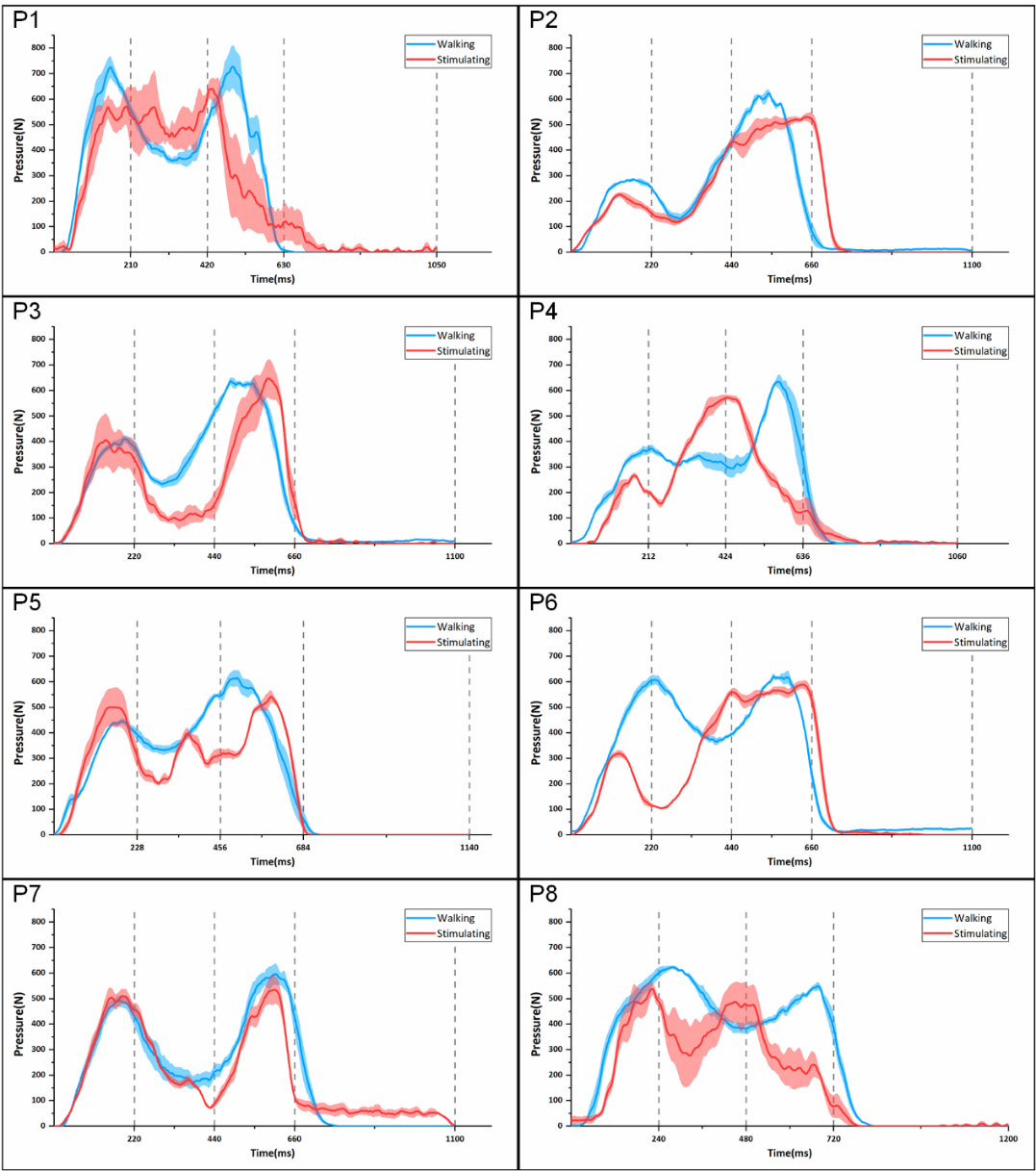


**Figure S1. The temporal change in foot sole pressure of partcipant1~partcipant8.** The blue line is the change during walking and the red is when stimulated by the stimulator. Each curve is the mean value with standard errors (shadow) from four separate pressure curves. The time axis is divided into four phases: Contact (0~Time*20%), Midstance (Time*20%~Time*40%), Propulsion (Time*40%~Time*60%), Swing (Time*60%~Time). (P1~P8: partcipant1~partcipant8).


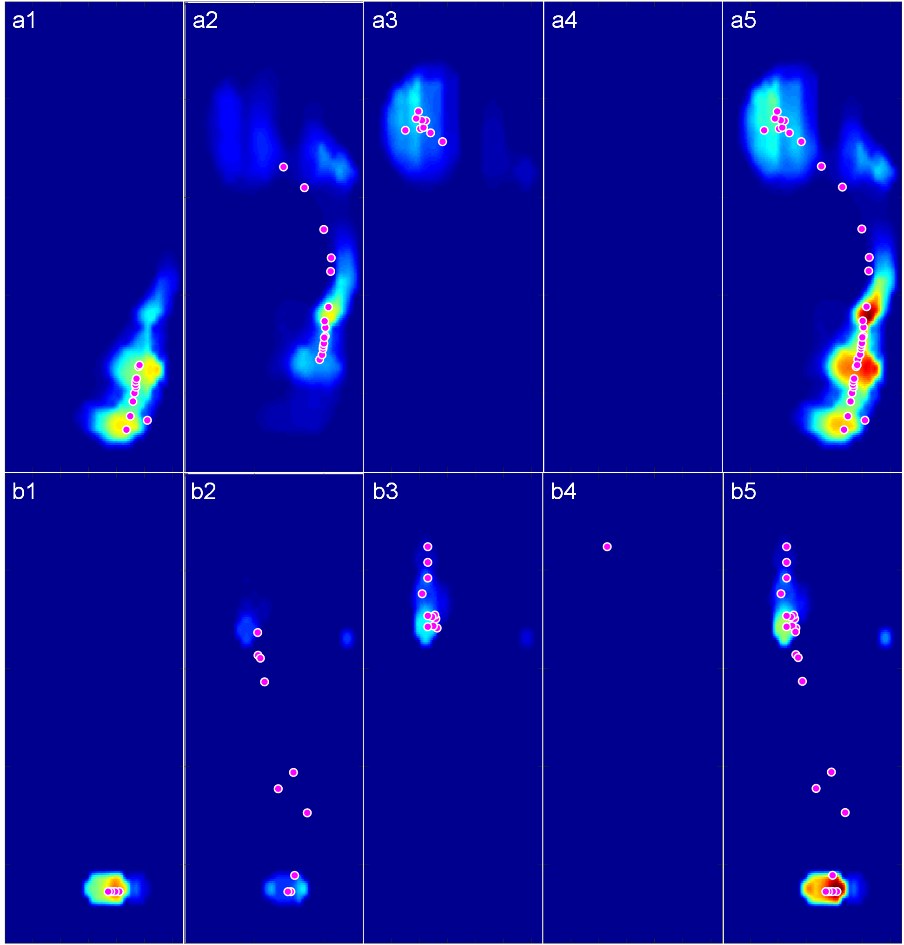


**Figure S2.** The partcipant1’s pressure maps with trajectory points of gravity center (Magenta circles with white edge). a1-a4: pressure maps in contact, midstance, propulsion and swing phase of a complete walking gait cycle. b1-b4: pressure maps in the four phases of a complete stimulating gait cycle. a5: pressure map of the whole walking gait cycle. b5: pressure map of the whole stimulating gait cycle.


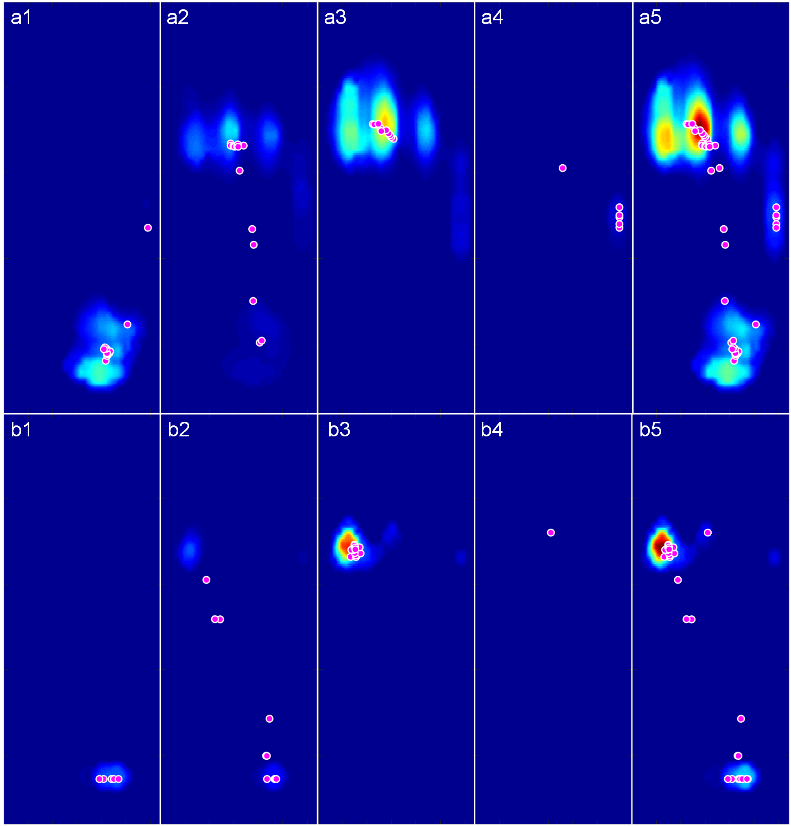


**Figure S3.** The partcipant2’s pressure maps.


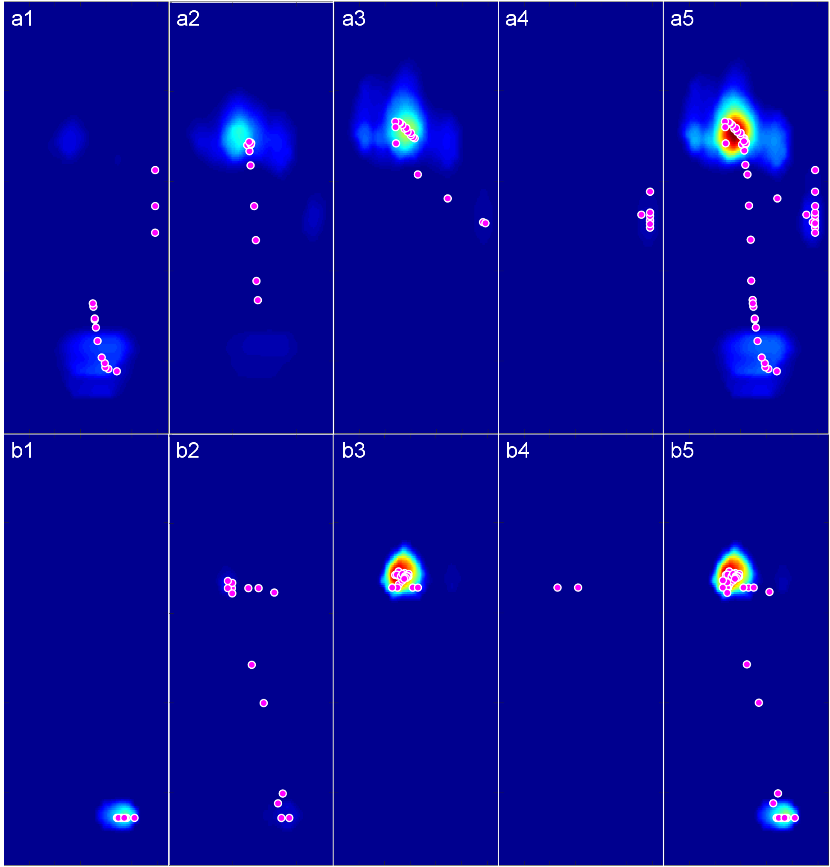


**Figure S4.** The partcipant3’s pressure maps.


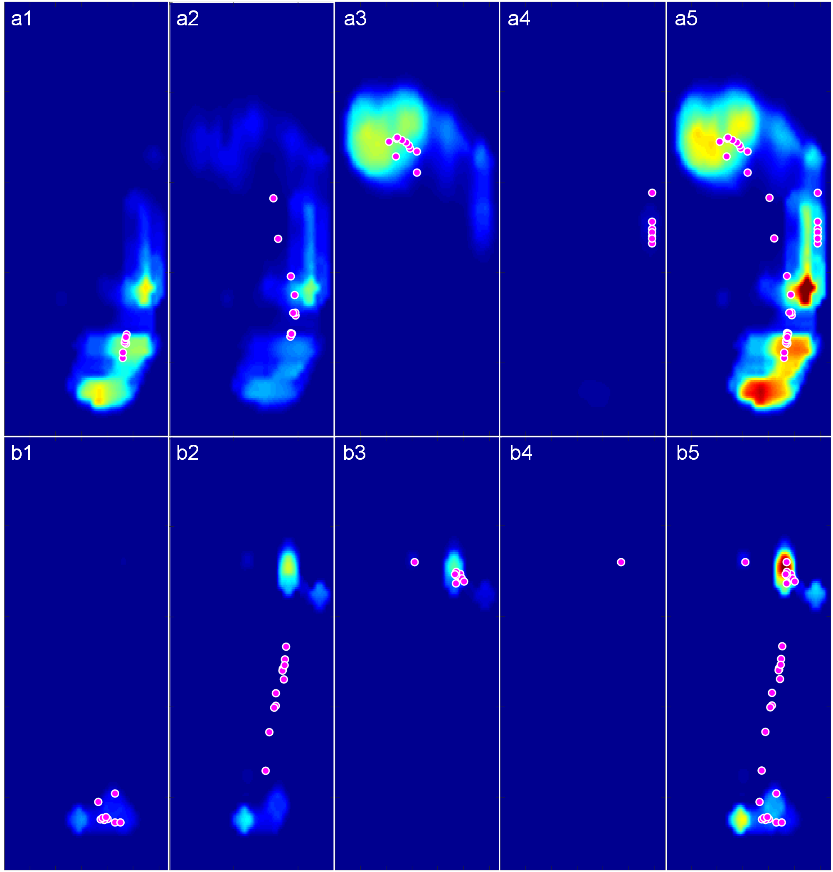


**Figure S5.** The partcipant4’s pressure maps.


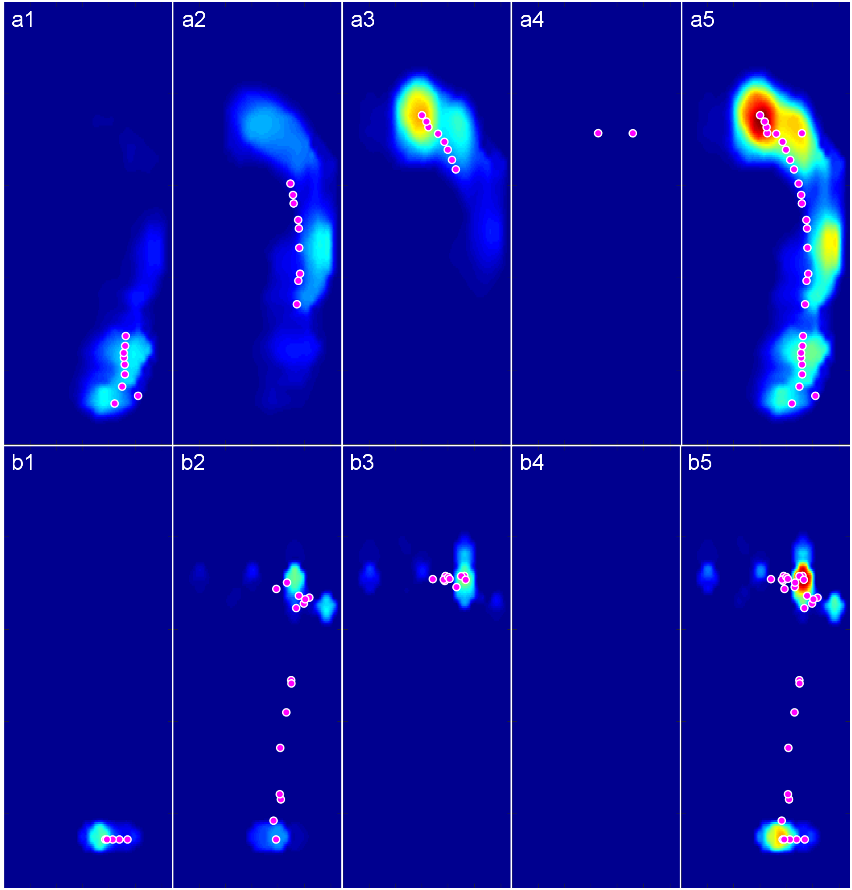


**Figure S6.** The partcipant5’s pressure maps.


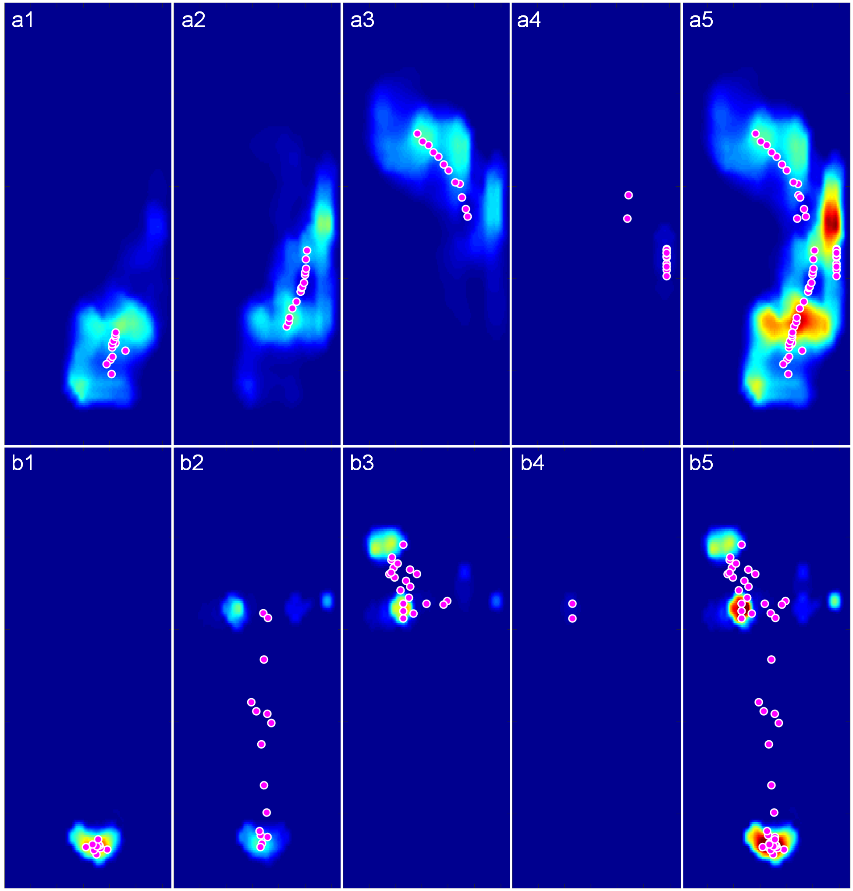


**Figure S7.** The partcipant6’s pressure maps.


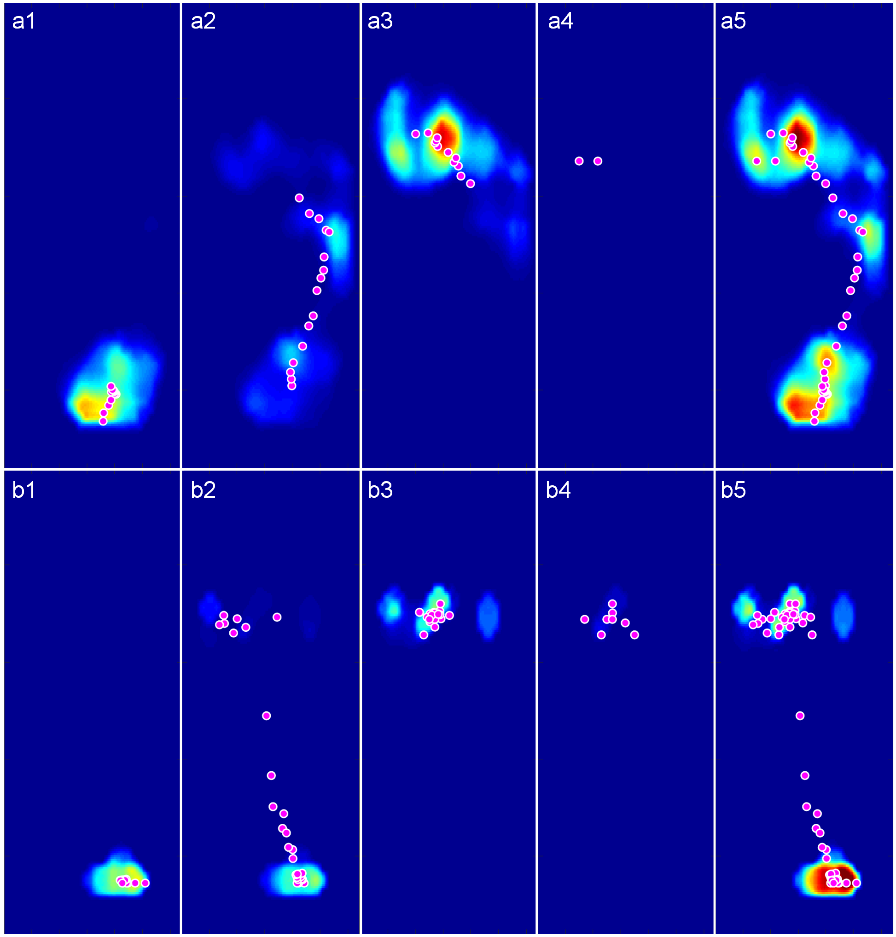


**Figure S8.** The partcipant7’s pressure maps.


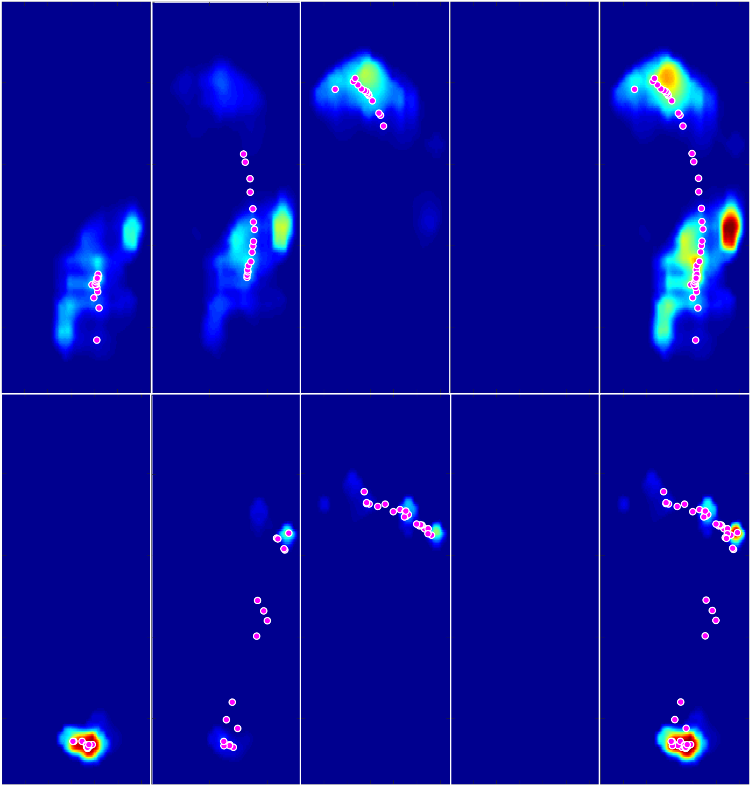


**Figure S9.** The partcipant8’s pressure maps.

**Table S1.** The Spearman’s correlation coefficients between each of nine participants' walking and stimulating pressure curves.

| Participant | 1 | 2 | 3 | 4 | 5 | 6 | 7 | 8 | 9 |
| --- | --- | --- | --- | --- | --- | --- | --- | --- | --- |
| r(W1S1) | 0.899 | 0.712 | 0.799 | 0.728 | 0.903 | 0.682 | 0.925 | 0.773 | 0.814 |
| r(W1S2) | 0.681 | 0.813 | 0.829 | 0.701 | 0.915 | 0.703 | 0.899 | 0.825 | 0.829 |
| r(W1S3) | 0.719 | 0.780 | 0.906 | 0.675 | 0.880 | 0.668 | 0.923 | 0.730 | 0.854 |
| r(W1S4) | 0.810 | 0.803 | 0.864 | 0.707 | 0.905 | 0.712 | 0.908 | 0.744 | 0.892 |
| r(W2S1) | 0.937 | 0.591 | 0.754 | 0.754 | 0.910 | 0.724 | 0.933 | 0.807 | 0.841 |
| r(W2S2) | 0.695 | 0.724 | 0.733 | 0.639 | 0.904 | 0.750 | 0.884 | 0.845 | 0.928 |
| r(W2S3) | 0.766 | 0.671 | 0.875 | 0.751 | 0.864 | 0.715 | 0.918 | 0.817 | 0.923 |
| r(W2S4) | 0.775 | 0.705 | 0.818 | 0.738 | 0.886 | 0.764 | 0.893 | 0.799 | 0.958 |
| r(W3S1) | 0.932 | 0.671 | 0.808 | 0.737 | 0.901 | 0.706 | 0.921 | 0.805 | 0.853 |
| r(W3S2) | 0.731 | 0.781 | 0.799 | 0.707 | 0.909 | 0.733 | 0.873 | 0.811 | 0.946 |
| r(W3S3) | 0.824 | 0.742 | 0.873 | 0.648 | 0.878 | 0.709 | 0.914 | 0.832 | 0.876 |
| r(W3S4) | 0.802 | 0.763 | 0.847 | 0.749 | 0.906 | 0.746 | 0.896 | 0.839 | 0.898 |
| r(W4S1) | 0.921 | 0.725 | 0.801 | 0.707 | 0.937 | 0.734 | 0.899 | 0.777 | 0.834 |
| r(W4S2) | 0.727 | 0.846 | 0.808 | 0.754 | 0.911 | 0.763 | 0.850 | 0.799 | 0.892 |
| r(W4S3) | 0.799 | 0.799 | 0.903 | 0.639 | 0.882 | 0.713 | 0.886 | 0.835 | 0.917 |
| r(W4S4) | 0.781 | 0.812 | 0.842 | 0.738 | 0.896 | 0.772 | 0.869 | 0.837 | 0.939 |
| Mean | 0.800 | 0.746 | 0.829 | 0.711 | 0.899 | 0.725 | 0.899 | 0.805 | 0.887 |
| SD | 0.084 | 0.067 | 0.049 | 0.041 | 0.018 | 0.029 | 0.023 | 0.034 | 0.046 |

r, The Spearman’s correlation coefficients; W1~W4, four walking pressure curves; S1~S4, four stimulating pressure curves; SD, Standard Deviation.
